# Inhibition of Cell Survival by Curcumin Is Associated with Downregulation of Cell Division Cycle 20 (Cdc20) in Pancreatic Cancer Cells

**DOI:** 10.3390/nu9020109

**Published:** 2017-02-04

**Authors:** Yu Zhang, Ying-bo Xue, Hang Li, Dong Qiu, Zhi-wei Wang, Shi-sheng Tan

**Affiliations:** 1 Department of Oncology, Guizhou People’s Hospital, Guizhou 550002, China; skyline_zyu@163.com (Y.Z.); xueyingbo0619@163.com (Y.-b.X.); lihang.sy@163.com (H.L.); qiudcds@126.com (D.Q.); 2 Department of Pathology, Beth Israel Deaconess Medical Center, Harvard Medical School, Boston, MA 02215, USA

**Keywords:** curcumin, Cdc20, pancreatic cancer, growth, invasion

## Abstract

Pancreatic cancer is one of the most aggressive human tumors in the United States. Curcumin, a polyphenol derived from the *Curcuma longa* plant, has been reported to exert its antitumor activity in pancreatic cancer. However, the molecular mechanisms of curcumin-mediated tumor suppressive function have not been fully elucidated. In the current study, we explore whether curcumin exhibits its anti-cancer function through inhibition of oncoprotein cell division cycle 20 (Cdc20) in pancreatic cancer cells. We found that curcumin inhibited cell growth, enhanced apoptosis, induced cell cycle arrest and retarded cell invasion in pancreatic cancer cells. Moreover, we observed that curcumin significantly inhibited the expression of Cdc20 in pancreatic cancer cells. Furthermore, our results demonstrated that overexpression of Cdc20 enhanced cell proliferation and invasion, and abrogated the cytotoxic effects induced by curcumin in pancreatic cancer cells. Consistently, downregulation of Cdc20 promoted curcumin-mediated anti-tumor activity. Therefore, our findings indicated that inhibition of Cdc20 by curcumin could be useful for the treatment of pancreatic cancer patients.

## 1. Introduction

Pancreatic cancer (PC) is one of the most highly aggressive malignancies in humans. According to the American Cancer Society, there will be 53,670 estimated new cases and 43,090 deaths due to PC in the United States in 2017 [1]. The routine treatments include surgery, radiation and chemotherapy. Although multiple-treatment approaches have been improved, the outcomes of PC patients are still poor. Because PC patients are diagnosed at a late stage and exhibit acquired drug resistance during chemotherapeutic treatment, the 5-year relative survival of PC is currently 7% [1]. Therefore, it is essential to find new agents to improve the treatment outcome in patients with PC.

Emerging evidence has supported that curcumin, a golden pigment extracted from turmeric, exhibits anti-tumor properties in various types of human cancers including PC [2,3,4,5]. For example, one study showed that curcumin inhibited interleukin 8 production in PC cells [6]. Moreover, it has been reported that curcumin suppressed cell proliferation and induced apoptosis through inhibition of nuclear factor-kappa B (NF-κB) and IkappaB (IκB) kinase in PC cells [7,8]. Further study found that curcumin synergistically potentiated the growth inhibitory and pro-apoptotic effects of celecoxib in PC cells [9]. Consistently, curcumin potentiates anticancer activity of gemcitabine via inhibition of proliferation, angiogenesis, and suppression of the NF-κB pathway in an orthotopic model of PC [10]. Notably, curcumin inhibited PC cell proliferation via downregulation of cyclooxygenase-2 (COX-2), epidermal growth factor receptor (EGFR) and extracellular signal regulated kinase (ERK1/2) signaling pathways [11]. Sahu et al. found that curcumin activated ataxia telangiectasia mutated (ATM)/check point kinase-1 (Chk1), leading to cell cycle arrest and apoptosis in human PC cells [12]. One study identified that Wilms’ tumor gene 1 (WT1) is one target of curcumin in PC cells [13]. Another study validated that curcumin inhibited constitutive signal transducer and activator of transcription 3 (STAT3) phosphorylation and down-regulated survivin/baculoviral inhibitor of apoptosis repeat-containing 5 (BIRC5) expression in PC cells [14]. Sun et al. reported that curcumin reversed the epithelial-mesenchymal transition through inhibition of the Hedgehog signaling pathway in PC cells [15]. Although these reports identified the molecular basis of curcumin-triggered tumor suppression, the underlying molecular mechanisms are still not clear.

Emerging evidence has revealed that cell division cycle 20 (Cdc20), an activator of the ligase anaphase-promoting complex/C (APC/C), plays an oncogenic role in tumorigenesis [16]. Higher expression of Cdc20 has been observed in a variety of human cancers and is correlated with poor prognosis [17,18,19]. For instance, patients with glioblastomas exhibited upregulation of Cdc20, while low-grade glioma patients have downregulation of Cdc20 [20]. Kim et al. found that Cdc20 was upregulated in high-grade squamous intraepithelial lesions and squamous cell carcinomas of the uterine cervix [17]. Similarly, increased Cdc20 expression is associated with development and progression of hepatocellular carcinoma [21]. Cdc20 and securin overexpression predicted short-team breast cancer survival [22]. Consistently, high Cdc20 expression is associated with poor prognosis in oral squamous cell carcinoma [18], gastric cancer [23], urothelial bladder cancer [24], colorectal cancer [19], non-small cell lung cancer [25], and pancreatic cancer [26]. In the current study, we explored whether Cdc20 exerts its oncogenic effects on cell growth, apoptosis, migration and invasion in PC cells. Moreover, we defined whether curcumin exerts its antitumor activity via downregulation of Cdc20 in PC cells. We found that curcumin inhibited the Cdc20 expression, leading to anti-cancer activity in PC cells. These results suggest that curcumin could be useful to inhibit the expression of Cdc20 in PC cells.

## 2. Materials and Methods

### 2.1. Cell Culture and Reagents

Human pancreatic cancer cell lines Patu8988 and Panc-1 were cultured in Dulbecco’s Modified Eagle Medium (DMEM) supplemented with 10% fetal bovine serum and 1% penicillin and streptomycin. The anti-Cdc20 antibody was purchased from Abcam Company (Cambridge, MA, USA). The anti-p21, anti-B-cell lymphoma 2 (Bcl-2), anti-B-cell lymphoma-extra large (Bcl-xL), anti-Bax, anti-caspase-3 antibodies were obtained from Cell Signaling Technology (Danvers, MA, USA). All secondary antibodies were purchased from Thermo Scientific (Waltham, MA, USA). The anti-β-actin, anti-Tubulin, and 3-(4,5-dimethyl-2-thiazolyl)-2,5-diphenyl-2-H-tetrazolium bromide (MTT) were purchased from Sigma-Aldrich (St. Louis, MO, USA). Inhibitor of APC/C (Apcin), a Cdc20 inhibitor, was purchased from BostonBiochem (Boston, MA, USA). Lipofectamine 2000 was purchased from Invitrogen (Waltham, MA, USA).

### 2.2. MTT Assay

The transfected PC cells were seeded at 5 × 10^3^ cells/well in 96-well plate for 24 h and treated with different concentrations of curcumin (chemical abstract service (CAS) number 458-37-7, 99.5% curcumin). Curcumin was dissolved in dimethyl sulfoxide (DMSO) to make a 30-mM stock solution and was directly added to the medium at different concentrations. Cells were treated with 0.1% DMSO as the control group. After 48 h and 72 h, MTT solution (5 mg/mL) was added to each well and incubated for 4 h at 37 °C. Then, 100 μL DMSO was added to dissolve the MTT-formazan crystals after the supernatant was absorbed. The absorption was measured by the microplate at 490 nm.

### 2.3. Cell Apoptosis Analysis

The transfected cells were cultured in six-well plate overnight and treated with curcumin for 48 h. Then, cells were harvested and washed with phosphate buffered saline (PBS), resuspended in 500 μL binding buffer with 5 μL propidium iodide (PI) and 5 μL fluorescein isothiocyanate (FITC)-conjugated anti-annexin V antibody. Apoptosis was measured with a FACScalibur flow cytometer (Becton, Dickinson Company, Franklin Lakes, NJ, USA). The Cell Death Detection ELISA Kit was also used for measuring apoptosis in PC cells treated with curcumin and Cdc20 short hairpin RNA (shRNA) according to the manufacturer’s protocol. Briefly, the cells were lysed after treatments, and the cell lysates were incubated in microtiter plate modules coated with antihistone antibody, and subsequently incubated with anti-DNA peroxidase followed by color development with 2,2′-azinobis-3-ethylbenzothiazoline-6-sulfonic acid (ABTS)™ substrate. The optical densities of the samples were determined by using the Ultra Multifunctional Microplate Reader at 405 nanometers (nm).

### 2.4. Cell Scratch Assays

The transfected cells were cultured in 6-well plate. After cells reached almost 100%, the supernatant was absorbed. Yellow pipette tips were used to scratch the cell surface. The cells were washed by PBS and treated with curcumin. The scratched area was photographed with a microscope at 0 h and 20 h, respectively. The experiment was repeated twice. The quantification of the results represents three times for cell scratch assays.

### 2.5. Cell Invasion Assay

Cell invasion assay was performed to explore the invasive activity of PC cells treated with curcumin or Cdc20 transfection or combination [27]. Briefly, transfected cells were seeded in the upper chamber with serum-free medium and 500 μL of complete medium was added in the under chamber with the same concentration of curcumin. After incubation for 24 h, the membrane of the chamber was strained with Giemsa and photographed with a microscope.

### 2.6. Transfection

Cells were seeded into 6-well plates and transfected with Cdc20 cDNA or Cdc20 shRNA or empty vector by lipofectamine 2000 following the instruction’s protocol. The transfected cells were subjected to further analysis as described under the results section.

### 2.7. Quantitative Real-Time Reverse Transcription-PCR (RT-PCR) Analysis

The total RNA was extracted with Trizol (Invitrogen, Carlsbad, CA, USA) and reversed-transcribed into cDNA by RevertAid First Strand cDNA Synthesis Kit. PCR were conducted by Power SYBR Green PCR Master Mix. The primers used in the PCR reaction were: Cdc20, forward primer (5′-GACCACTCCTAGCAAACCTGG-3′) and reverse primer (5′-GGGCGTCTGGCTGTTTTCA-3′); Glyceraldehyde-3-phosphate dehydrogenase (GAPDH), forward primer (5′-ACCCAGAAGACTGTGGATGG-3′) and reverse primer (5′-CAGTGAGCTTCCCGTTCA G-3′).

### 2.8. Western Blotting Analysis

The harvested cells were washed by PBS and treated with protein lysis buffer. The BCA Protein Assay kit (Thermo Scientific, Waltham, MA, USA) was used to measure the concentrations of the proteins. Protein samples were separated by electrophoresis in sodium dodecyl sulfate (SDS)-polyacrylamide gel and then transferred onto a polyvinylidene difluoride (PVDF) membrane, and then incubated with primary antibody at 4 °C overnight. Then, cells were washed with Tris-buffered saline and Tween 20 (TBST) for three times and incubated with second antibody at room temperature for 1 h. Then the expression of protein was determined by enhanced luminal-based chemiluminescent (ECL) assay.

### 2.9. Statistical Analysis

All statistical analyses were measured by GraphPad Prism 5.0 (Graph Pad Software, La Jolla, San Diego, CA, USA). ANOVA assay was used to evaluate statistical significance. Results were presented as means ± SD. *p* < 0.05 was considered as statistically significant.

## 3. Results

### 3.1. Curcumin Inhibited the Expression of Cdc20

**Figure 1:**
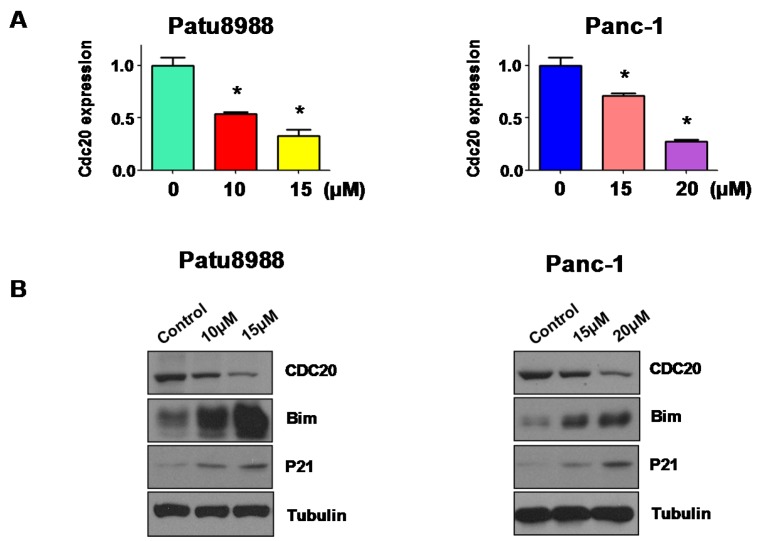

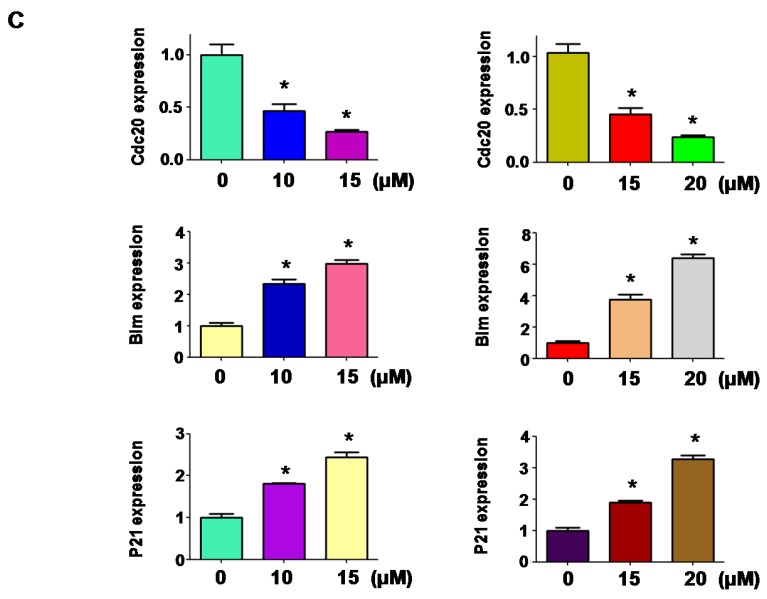
Curcumin decreased cell division cycle 20 (Cdc20) expression at RNA and protein levels. (**A**) The Cdc20 mRNA expression was measured by real-time reverse transcription-PCR (RT-PCR) in PC cells treated with curcumin. * *p* < 0.05, vs. control; (**B**) The expression of Cdc20, Bim, and p21 was detected using Western blotting analysis in pancreatic cancer (PC) cells after curcumin treatment; (**C**) Quantitative results are illustrated for panel B. * *p* < 0.05, compared to the control.

Multiple studies have shown that curcumin inhibited cell growth in PC cells. Since Cdc20 has been considered to play an important oncogenic role in pancreatic tumorigenesis, we tested whether curcumin could suppress the expression of Cdc20 in PC cells. Real-time (RT)-PCR) was performed to measure the mRNA level of Cdc20 in PC cells treated with curcumin. Our RT-PCR results showed that curcumin treatment significantly decreased Cdc20 mRNA level in both Patu8988 and Panc-1 cells (Figure 1A). To determine whether curcumin could decrease the Cdc20 protein level, western blotting analysis was conducted to measure the Cdc20 protein expression in PC cells after curcumin treatment. We found that curcumin remarkably reduced the Cdc20 protein level in PC cells (Figure 1B,C). It is known that Bim and p21 are two downstream targets of Cdc20. Indeed, we observed that curcumin treatment led to upregulation of Bim and p21 in both PC cells (Figure 1B,C). These findings revealed that curcumin inhibited Cdc20 expression in PC cells.

### 3.2. Overexpression of Cdc20 Decreased Curcumin-Induced Cell Growth Inhibition

**Figure 2:**
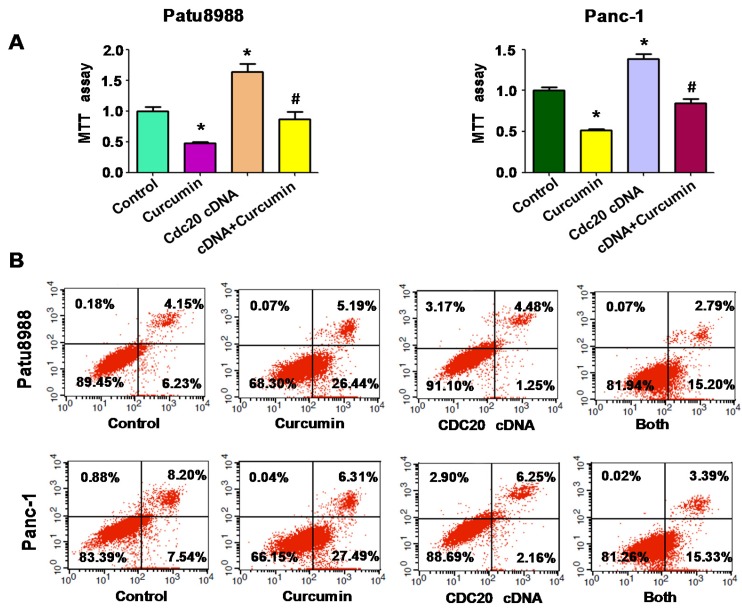

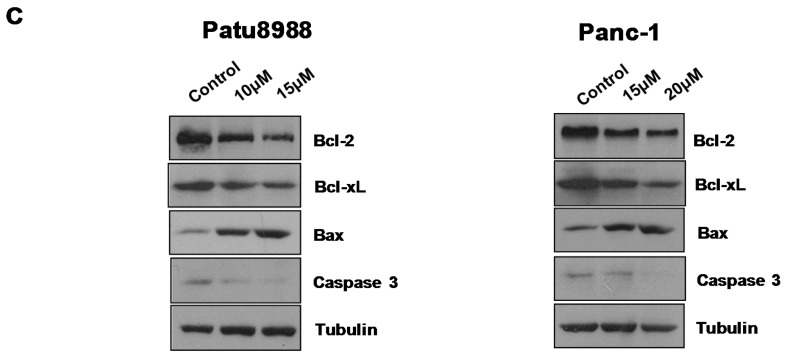
Overexpression of Cdc20 decreased curcumin-induced cell growth inhibition and apoptosis. (**A**) A 3-(4,5-dimethyl-2-thiazolyl)-2,5-diphenyl-2-H-tetrazolium bromide (MTT) assay was performed to measure the cell growth in PC cells with Cdc20 cDNA transfection in combination with curcumin treatment. * *p* < 0.05, compared with control; **^#^** *p* < 0.05 compared with curcumin treatment or Cdc20 cDNA transfection alone; (**B**) Cell apoptosis was determined by flow cytometry in PC cells treated with curcumin plus Cdc20 cDNA transfection; (**C**) Western blotting analysis was performed to measure the expression of Bcl-2 family and caspase-3 in PC cells after curcumin treatment.

To explore whether curcumin-mediated cell growth inhibition is through suppression of Cdc20 in PC cells, Patu8988 and Panc-1 cells were transfected with Cdc20 cDNA or empty vector as control group. Our MTT results showed that overexpression of Cdc20 significantly enhanced cell growth in both PC cell lines (Figure 2A). Consistently, curcumin inhibited cell growth in Patu8988 and Panc-1 cells (Figure 2A). Importantly, overexpression of Cdc20 rescued cell growth suppression by curcumin treatment in PC cells (Figure 2A). Our data suggest that curcumin exerts its inhibition of cell growth via downregulation of Cdc20 in PC cells.

### 3.3. Overexpression of Cdc20 Abrogated Curcumin-Triggered Cell Apoptosis

It is known that curcumin treatment leads to induction of cell apoptosis in PC cells. In line with this concept, we found that curcumin induced cell apoptosis in both PC cell lines (Figure 2B). Moreover, we found that curcumin enhanced apoptosis via inhibition of Bcl-2, Bcl-xL and upregulation of Bax and Caspase-3 in PC cell lines (Figure 2C). One study has revealed that Cdc20 inhibited cell apoptosis via degradation of Bim in human cancer cells [28]. Indeed, we found that overexpression of Cdc20 suppressed cell apoptosis in PC cells (Figure 2B). Strikingly, Cdc20 cDNA transfection abrogated curcumin-induced cell apoptosis in PC cells (Figure 2B). Therefore, curcumin-triggered cell apoptosis is partly through downregulation of Cdc20 in PC cells.

### 3.4. Overexpression of Cdc20 Retarded Curcumin-Mediated Cell Motility Inhibition

**Figure 3:**
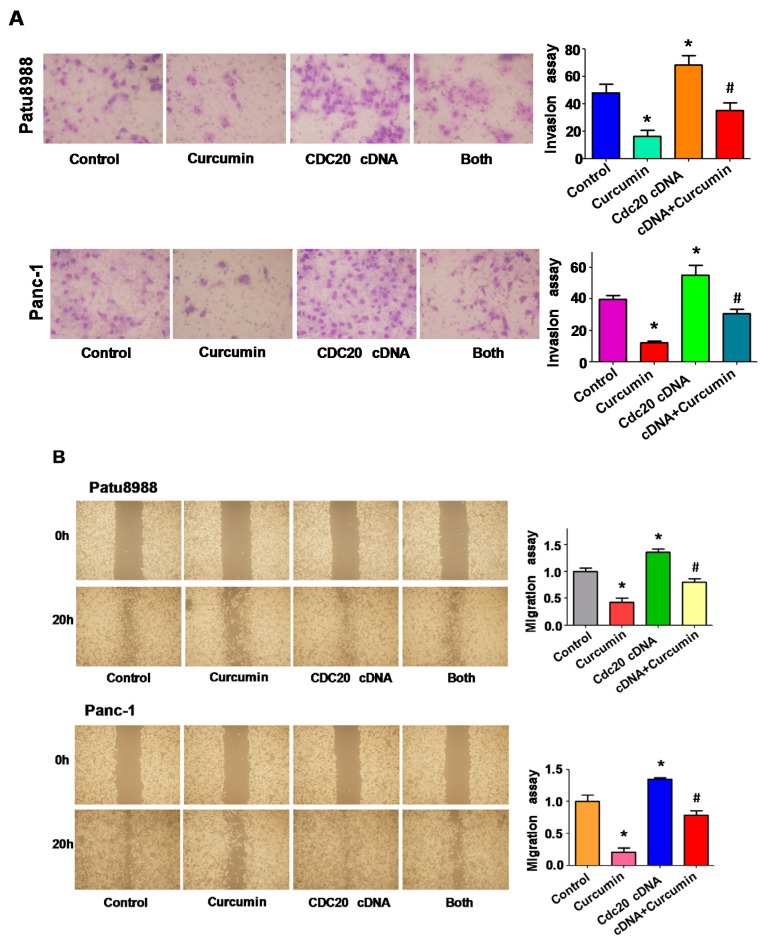
Overexpression of Cdc20 retarded curcumin-mediated cell motility inhibition. (**A**) Left panel, invasion assay was conducted in PC cells after Cdc20 cDNA transfection and curcumin treatment. Right panel, quantitative results are illustrated for left panel. * *p* < 0.05, compared with control; **^#^** *p* < 0.05 compared with curcumin treatment or Cdc20 cDNA transfection alone; (**B**) Left panel, the wound healing assay was performed to measure the cell migration in PC cells after Cdc20 cDNA transfection and curcumin treatment. Both: Cdc20 CDNA + curcumin. Right panel, quantitative results are illustrated for left panel. * *p* < 0.05, compared with control; **^#^** *p* < 0.05 compared with curcumin treatment or Cdc20 cDNA transfection alone.

Next, to investigate whether Cdc20 could govern cell motility in PC cells, the Transwell chambers assay was used to measure the cell invasion in PC cells treated with curcumin and Cdc20 cDNA transfection. The results from Transwell assays showed that curcumin significantly reduced the cell invasion in PC cells (Figure 3A). Overexpression of Cdc20 promoted cell invasion in both PC cells (Figure 3A). Notably, overexpression of Cdc20 retarded curcumin-mediated cell invasion inhibition (Figure 3A). To further validate the function of curcumin in cell motility, the wound healing assay was performed in PC cells with Cdc20 cDNA transfection and curcumin treatment. We observed that curcumin remarkably inhibited cell migration in PC cells (Figure 3B). Overexpression of Cdc20 promoted cell migration in PC cells (Figure 3B). Importantly, overexpression of Cdc20 blocked curcumin-induced cell migration inhibition (Figure 3B). These findings suggest that curcumin inhibited cell motility via down-regulation of Cdc20 in PC cells.

### 3.5. Overexpression of Cdc20 Abrogated Activation of p21 and Bim by Curcumin

**Figure 4:**
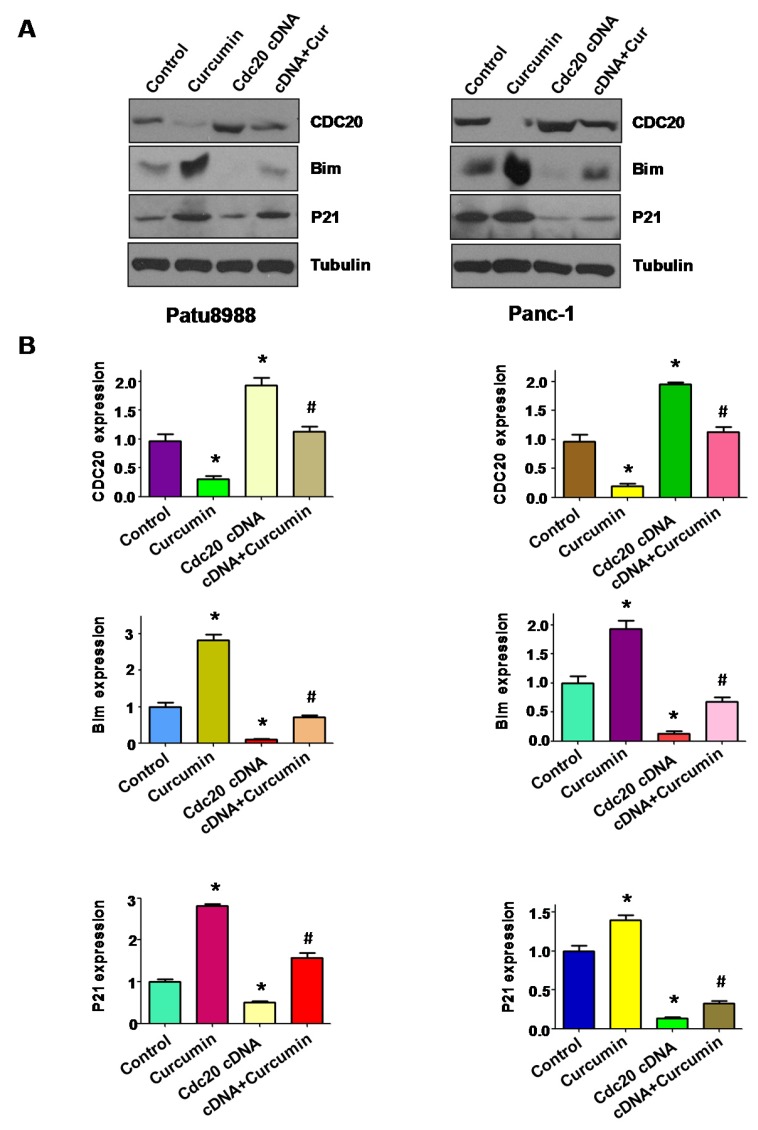
The expression of Cdc20, Bim and p21 was measured in PC cells. (**A**) The expression of Cdc20 and its targets Bim and p21 was measured by western blotting in PC cells with Cdc20 cDNA transfection and curcumin treatment (15 μM curcumin for patu8988 and 20 μM curcumin for Panc-1); (**B**) Right panel: Quantitative results are illustrated for left panel. * *p* < 0.05, compared with control; **^#^** *p* < 0.05 compared with curcumin treatment or Cdc20 cDNA transfection.

It has been well documented that Cdc20 controlled the ubiquitin-mediated degradation of p21, leading to regulation of cell growth [29]. Therefore, we investigated whether overexpression of Cdc20 by its cDNA could block the upregulation of p21 by curcumin treatment. In keeping with this, we found that overexpression of Cdc20 abrogated expression of p21 by curcumin in PC cells (Figure 4A,B). One study demonstrated that Bim is one key target of Cdc20, which is involved in regulation of cell apoptosis [28]. Mechanistically, we found that upregulation of Cdc20 abrogated activation of Bim induced by curcumin in PC cells (Figure 4A,B). These results provide the molecular insight that curcumin exhibits anti-tumor activity through downregulation of Cdc20 and its downstream targets including p21 and Bim.

### 3.6. Downregulation of Cdc20 Promotes Curcumin-Mediated Cell Growth Inhibition and Apoptosis

**Figure 5:**
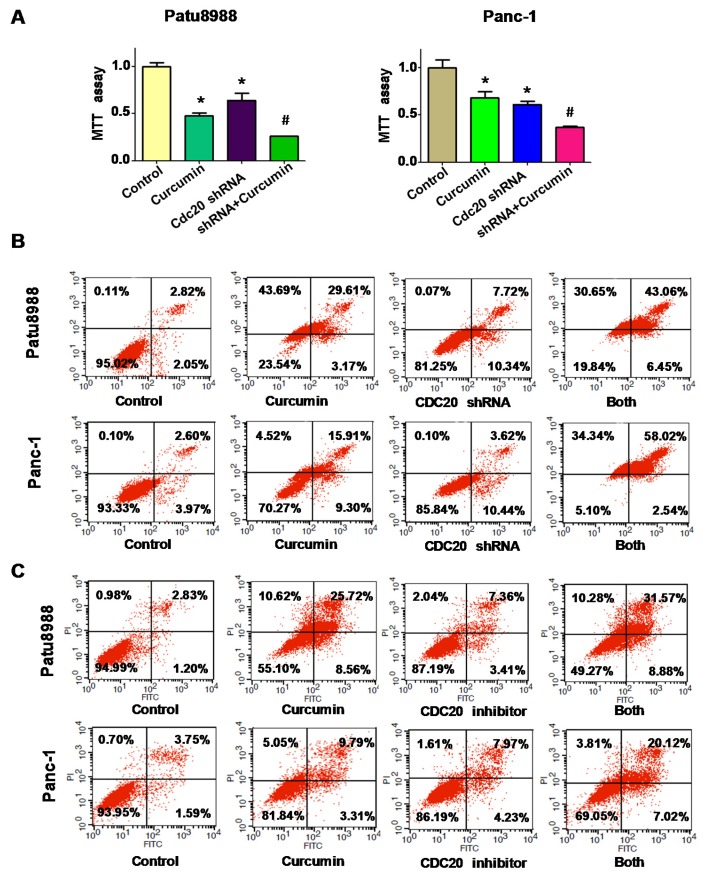
The effect of Cdc20 downregulation on cell growth and apoptosis. (**A**) MTT assay was conducted to determine the effect of Cdc20 shRNA in combination with curcumin treatment on PC cell growth. * *p* < 0.05, compared with control; **^#^** *p* < 0.05 compared with curcumin treatment or Cdc20 shRNA transfection; (**B**) Apoptosis was detected by flow cytometry in PC cells with Cdc20 shRNA transfection and curcumin treatment. Both: curcumin + Cdc20 shRNA. C. Apoptosis was analyzed by flow cytometry in PC cells with Cdc20 inhibitor (50 μM Apcin) and curcumin treatment. Both: curcumin + Cdc20 inhibitor. PI: propidium iodide; FITC: fluorescein isothiocyanate.

To more deeply determine whether curcumin mediated cell growth suppression via downregulation of Cdc20, MTT assay was conducted in PC cells treated with Cdc20 shRNA and curcumin. Consistent with the oncogenic role of Cdc20, we found that downregulation of Cdc20 caused cell growth inhibition (Figure 5A). Moreover, our results showed that downregulation of Cdc20 expression significantly suppressed cell growth induced by curcumin (Figure 5A). Furthermore, downregulation of Cdc20 by its shRNA induced cell apoptosis in PC cells (Figure 5B). Accordingly, Cdc20 shRNA transfected cells were significantly more sensitive to curcumin-induced apoptosis in PC cells (Figure 5B). To further validate our results, apcin as a Cdc20 inhibitor was used to determine whether inhibition of Cdc20 by its inhibitor promoted cell apoptosis in PC cells. We observed that 50 μM of apcin induced apoptosis in both PC cells (Figure 5C). The results from ELISA for histone/DNA fragment analysis also confirmed that inhibition of Cdc20 enhanced curcumin-triggered cell apoptosis (Supplementary Materials Figure S1).

### 3.7. Downregulation of Cdc20 by shRNA Promotes Curcumin-Induced Cell Invasion Inhibition

**Figure 6:**
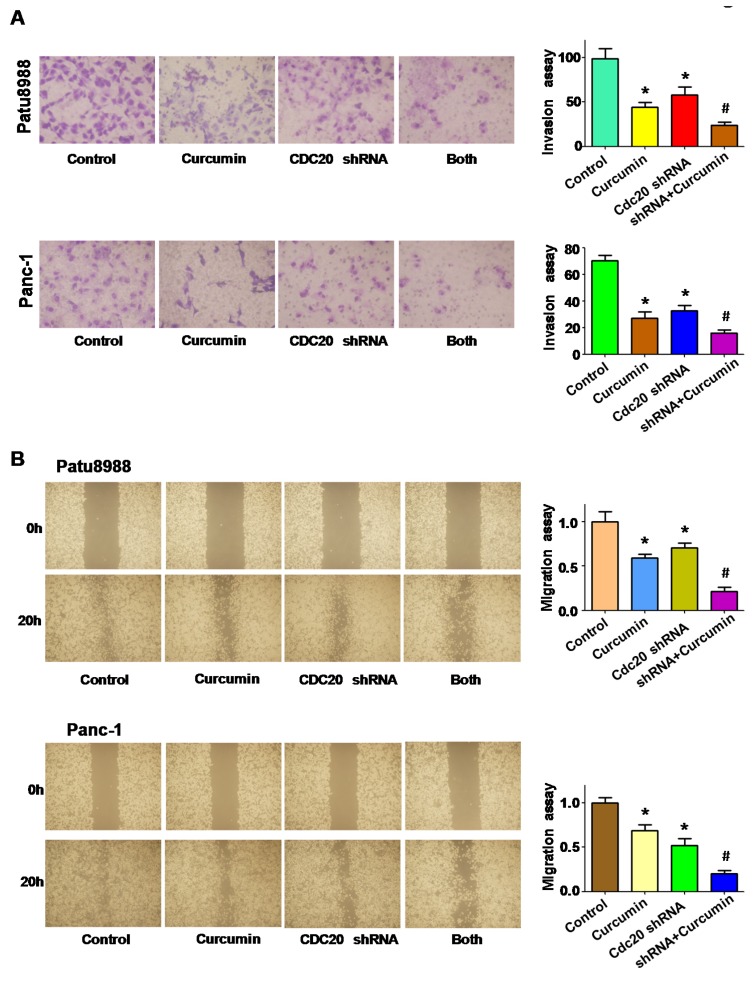
The effect of Cdc20 downregulation on cell migration, and invasion. (**A**) Invasion assay was conducted in PC cells after Cdc20 shRNA transfection and curcumin treatment; (**B**) The wound healing assay was used to measure the cell migration in PC cells after Cdc20 shRNA transfection and curcumin treatment. Both: curcumin + Cdc20 shRNA. * *p* < 0.05, vs. control; **^#^** *p* < 0.05 vs. curcumin treatment or Cdc20 shRNA transfection.

**Figure 7:**
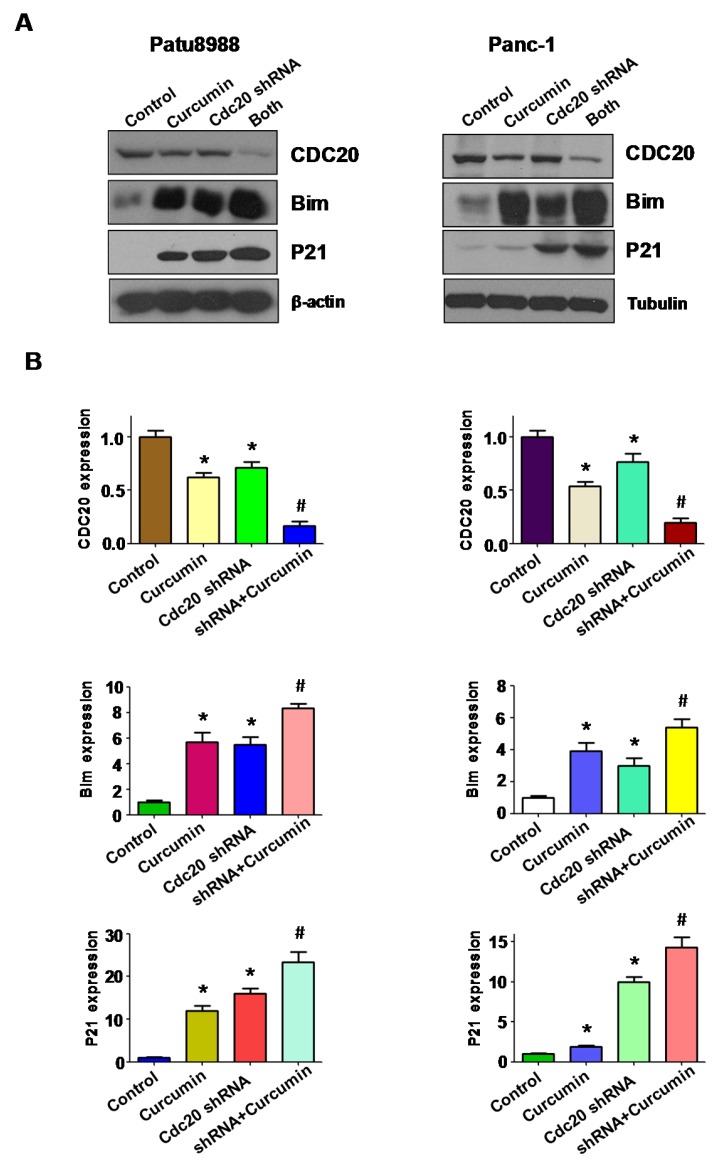
The expression of Cdc20, Bim and p21 was measured in PC cells. (**A**) The expression of Cdc20 and its targets Bim and p21 was measured by Western blotting in PC cells after Cdc20 shRNA transfection and curcumin treatment; (**B**) Quantitative results are illustrated for panel A. * *p* < 0.05, compared with control; **^#^** *p* < 0.05 compared with curcumin treatment or Cdc20 shRNA transfection.

To further validate whether curcumin exhibited its anti-tumor invasion via down-regulation of Cdc20, Tanswell chamber assays and wound healing assays were conducted in PC cells treated with curcumin and Cdc20 shRNA transfection. We found that downregulation of Cdc20 retarded cell invasion in both PC cell lines (Figure 6A). Concurrently, downregulation of Cdc20 in combination of curcumin treatment led to inhibition of cell invasion to a greater degree compared with curcumin treatment alone or shRNA transfection alone (Figure 6A). Consistently, the wound healing assay showed similar results, suggesting that downregulation of Cdc20 promoted curcumin-mediated cell migration inhibition (Figure 6B). Intriguingly, our results revealed that Cdc20 shRNA increased the expression of Bim and p21 expression (Figure 7A,B). Importantly, curcumin plus Cdc20 shRNA increased p21 and Bim expression to greater degree compared to curcumin alone or shRNA transfection alone (Figure 7A,B).

## 4. Discussion

Studies have defined the molecular mechanism of curcumin-induced tumor suppression. For example, curcumin induced cell death through reduction of the inhibitors of apoptosis in PC cells [30]. Moreover, curcumin induced cell apoptosis via inhibition of forkhead box O1 and PI3K/Akt pathway [31]. Our previous study showed that curcumin exerted its anti-tumor activity via down-regulation of Yes-associated protein and transcriptional co-activator with PDZ binding motif (YAP/TAZ) expression in PC cells [32]. Moreover, it has been reported that curcumin inhibited cell growth and invasion via suppression of Skp2 [33]. Cao et al. validated that curcumin inhibited H_2_O_2_-induced invasion and migration through inactivation of ERK/NF-κB pathway in PC [34]. Furthermore, curcumin was found to inhibit hypoxia-induced epithelial-mesenchymal transition (EMT) in PC cells via inhibition of the hedgehog signaling pathway [35]. In our current study, we found that curcumin suppressed cell growth and triggered cell cycle arrest and induced apoptosis in PC cells. We further demonstrated that curcumin retarded cell migration and invasion in PC cells. Mechanistically, curcumin exerts its anti-tumor functions through down-regulation of Cdc20 expression in PC cells. These results indicated that curcumin could be an effective agent for the treatment of PC. Targeting numerous of molecules by curcumin could represent a novel approach for treating PC patients.

Robust evidence has demonstrated that Cdc20 plays an oncogenic role in human tumorigenesis. Mechanistically, overexpression of Cdc20 caused impairment of the spindle assembly checkpoint and aneuploidization in oral cancer [36]. Another study revealed that Cdc20 suppressed apoptosis through targeting Bim for ubiquitination and destruction [28]. Additionally, Cdc20 induced degradation of conductin-regulated Wnt/β-catenin signaling for maximal activity during G1/S [37]. Furthermore, Cdc20 could be negatively governed by p53 [38]. Notably, depletion of Cdc20 by its shRNA inhibited the cell growth and induced cell cycle arrest [39]. In line with this report, our study showed that overexpression of Cdc20 enhanced cell growth, whereas depletion of Cdc20 inhibited cell growth in PC cells. Moreover, depletion of Cdc20 led to enhancement of the cytotoxicity of paclitaxel and increased the effect of irradiation against PC cells [39]. Similarly, silencing the expression of Cdc20 by liposomally-encapsulated Cdc20 siRNA inhibited tumor growth via induction of apoptosis of the tumor endothelial cells [40].

It is important to discover the inhibitors of Cdc20 for the treatment of human cancers. Tosyl-l-arginine methyl ester (TAME) reduced Cdc20 association with the APC, leading to inhibition of APC E3 ligase activity [41]. Strikingly, pro-TAME with cell permeable activity disrupted the APC-Cdc20/Cdh1 interaction to inhibit APC activation [41]. Furthermore, APC inhibitors (apsin) could bind Cdc20 and block substrate recognition and inhibit the ubiquitination of Cdc20 substrates [42]. Jiang et al. found that ganodermanontriol exerts its inhibitory effects of cell growth and invasiveness via the downregulation of Cdc20 in breast cancer cells [43]. One study showed that withaferin A modulated the spindle assembly checkpoint by degradation of Mad2-Cdc20 complex in colorectal cancer cells [44]. Zhang et al. identified that compound 331 selectively induced glioma cell death through downregulation of Cdc20 [45]. Recently, rottlerin was found to downregulate the expression of Cdc20 in glioma cells [46]. Our current study showed that curcumin inhibited the expression of Cdc20. 

## 5. Conclusions

In the current study, our results demonstrated that curcumin suppressed Cdc20 expression in PC cells, suggesting that curcumin could be a promising agent for treating human PC cancer. However, further investigation in the future is required to explore whether curcumin sensitizes chemotherapeutic drugs via downregulation of Cdc20 in PC patients.

## Supplementary Materials

The following are available online at http://www.mdpi.com/2072-6643/9/2/109/s1, Figure S1: Cell apoptosis was measured with the histone/DNA fragment analysis by using an enzyme-linked immune adsorbent assay.
