# Supplementary material for: Inhibition of Cell Survival by Curcumin Is Associated with Downregulation of Cell Division Cycle 20 (Cdc20) in Pancreatic Cancer Cells
Source: Nutrients. 2017 Feb 4;9(2):109. doi: 10.3390/nu9020109 (PMC5331540; doi:10.3390/nu9020109)
Supplement: Supplementary file 1 [file nutrients-09-00109-s001.docx]

Supplementary Materials: Inhibition of Cell Survival by Curcumin Is Associated with Down-Regulation of Cdc20 in Pancreatic Cancer Cells

Yu Zhang, Ying-bo Xue, Hang Li, Dong Qiu, Zhi-wei Wang and Shi-sheng Tan


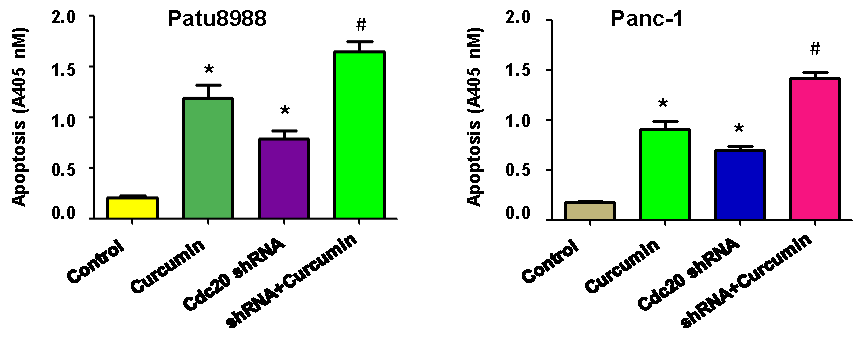


B

A

**Figure S1.** Cell apoptosis was measured with the histone/DNA fragment analysis by using an enzyme-linked immune adsorbent assay. (**A**) Apoptosis was determined by ELISA in Patu8988 cells treated with curcumin or Cdc20 shRNA or combination. * *p* < 0.05, compared with control; **^#^** *p* < 0.05 compared with curcumin treatment or Cdc20 shRNA transfection; (**B**) Apoptosis was measured by ELISA in Panc-1 cells treated with curcumin or Cdc20 shRNA or combination. * *p* < 0.05, compared with control; **^#^** *p* < 0.05 compared with curcumin treatment or Cdc20 shRNA transfection.
